# Precise colloids with tunable interactions for confocal microscopy

**DOI:** 10.1038/srep14635

**Published:** 2015-09-30

**Authors:** Thomas E. Kodger, Rodrigo E. Guerra, Joris Sprakel

**Affiliations:** 1 School of Engineering and Applies Sciences, Harvard University, Cambridge, 02138, USA; 2 Department of Physics, Harvard University, Cambridge, MA, 02138, USA; 3 Physical Chemistry and Soft Matter, Wageningen University, Dreijenplein 6, 6703 HB, Wageningen, the Netherlands

## Abstract

Model colloidal systems studied with confocal microscopy have led to numerous insights into the physics of condensed matter. Though confocal microscopy is an extremely powerful tool, it requires a careful choice and preparation of the colloid. Uncontrolled or unknown variations in the size, density, and composition of the individual particles and interactions between particles, often influenced by the synthetic route taken to form them, lead to difficulties in interpreting the behavior of the dispersion. Here we describe the straightforward synthesis of copolymer particles which can be refractive index- and density-matched simultaneously to a non-plasticizing mixture of high dielectric solvents. The interactions between particles are accurately tuned by surface grafting of polymer brushes using Atom Transfer Radical Polymerization (ATRP), from hard-sphere-like to long-ranged electrostatic repulsion or mixed charge attraction. We also modify the buoyant density of the particles by altering the copolymer ratio while maintaining their refractive index match to the suspending solution resulting in well controlled sedimentation. The tunability of the inter-particle interactions, the low volatility of the solvents, and the capacity to simultaneously match both the refractive index and density of the particles to the fluid opens up new possibilities for exploring the physics of colloidal systems.

Colloidal systems are used to explore the physics of condensed matter in real-time and space; observations of the behavior of colloidal particles have led to unprecedented insight into phenomena as varied as crystal nucleation^1^ and melting^2^, defect transport^3^, glass formation^4^,^5^, wetting and capillary phenomena^6^, and self-assembly and specific bonding^7^. Unlike their atomic counterparts, the structure, dynamics, and mechanical properties of these dispersions are accessible by optical microscopy and light scattering. However, practical limitations of each of these techniques and the design of individual experiments makes control over the physical properties of the constituent particles essential. Optical microscopy, which reveals the real-space structure of colloids, and light scattering, which elucidates the structure and dynamics in reciprocal space, rely on the precise control of the size and refractive index of the observed particles: as the choice of particle size influences the relative time and length scales available to the experiment, and the careful matching of the refractive indexes of the particles and suspending fluid minimizes the effects of optical aberrations and multiple scattering. To study the evolution of samples over long time scales it is essential the evaporation of the solvent is minimal; this is particularly important during rheological measurements in which the suspensions are exposed to the environment. Gravitational stresses, which cannot be ignored for micrometer scale particles, result in density gradients and sedimentation that strongly affect material properties such as crystal nucleation rates^8^. These detrimental effects of gravity can be minimized by matching the density of the particles to that of the suspending fluid: thus enabling the study of equilibrium, bulk behavior. Nevertheless, a well controlled density mismatch can be desirable^6^,^9^,^10^, for example when templating specific crystalline structures on a patterned surface^11^,^12^,^13^.

In addition to the physical properties of individual particles and the surrounding fluid, the structure and dynamics of colloidal suspensions are also determined by the forces that particles exert on each other^14^,^15^,^16^. The simplest interaction between solid particles is that of volume exclusion; in this so-called hard-sphere limit, particles are assumed to be impenetrable, and the phase behavior is governed by particle volume fraction alone. Two commonly used experimental systems that exhibit such a hard-sphere interaction potential and may be refractive index- and density-matched are poly (methyl methacrylate) (PMMA) microspheres stabilized by a brush of poly(hydroxystearic acid) (PHSA-PMMA) dispersed in a mixture of low dielectric organic solvents^15^,^16^ and stearylated-silica dispersed in halogenated fluids^17^. However, despite the low polarity of these solvents, trace amounts of ionizable impurities dissolved in these oils can charge particle surfaces in ways that are hard to predict and control^16^,^18^: muddling otherwise purely hard-sphere interactions. Moreover, the most commonly used hard-sphere system, PHSA-PMMA, is often dispersed in fluid mixtures in which one or more of its components swells the polymer that composes the particles, sometimes by as much as several tens of percents. This may be expected to significantly lower the glass transition temperature, softening the particles, and changing their properties over time^16^. Finally, while this particular system has proven invaluable as a colloidal model system, it synthesis is notoriously difficult to reproduce. More elaborate inter-particle interactions can be engineered by adding polymers or surfactants to the fluid, and by modifying the surfaces of the particles themselves. Ionizable moieties or polymer brushes grafted onto the surface of colloidal particles give rise to additional parameters to control the phase behavior of the suspension^19^,^20^,^21^,^22^. For example, suspensions of like charged colloids can organize into low density crystalline phases with symmetries not accessible for hard spheres^19^. By contrast, mixtures of colloidal particles with opposite charges can form structured colloidal gels^21^,^23^ and binary crystalline superlattices^24^,^25^. It remains a challenge to synthesize colloidal particles with controllable interaction potentials and predictable yet flexible physical properties such as surface charge, refractive index and density.

In this paper, we describe the synthesis of a colloidal model system composed of monodisperse particles that can be simultaneously refractive index- and density-matched to mixtures of non-hazardous, polar solvents that do not plasticize the particles whose surfaces may be modified with polymer brushes grafted using Atom Transfer Radical Polymerization (ATRP). We produce monodisperse particles of poly(trifluoroethyl methacrylate -*co*- *t*-butyl methacrylate) by means of dispersion polymerization using commercially available ingredients. Polymer brushes grafted from the particle surface using surface-initiated ATRP^26^ mediate forces between particles and may be sensitively tuned from hard-sphere-like to soft and long-ranged repulsive. Mixtures of these particles, functionalized with oppositely charged brushes show reversible charge-driven aggregation^21^,^22^. The refractive index and density of these particles can be tuned by altering the copolymer ratio. Finally, we prepare these copolymer particles containing a fluorescent core and a non-fluorescent shell that enhances the accuracy of particle locating in densely-packed 3D confocal experiments^27^,^28^.

## Results and Discussion

The experimental limitations of many colloidal systems are often a direct consequence of the material that composes the dispersed particles. The relatively low refractive index, *n*, of silica particles can be matched to a wide variety of solvent mixtures, but matching their relatively high density, *ρ*, is challenging. Conversely, the low density of polystyrene particles can be matched, for example, by H_2_O/D_2_O mixtures, but their *n* is too high to be matched, hindering the study of concentrated systems by optical methods. By contrast, methacrylate polymers exhibit refractive indices and densities which can be simultaneously matched. This is traditionally done in apolar, halogenated solvents, which swell and plasticize the particles, and in which charge interactions are difficult to control^16^.

**Figure 1:**
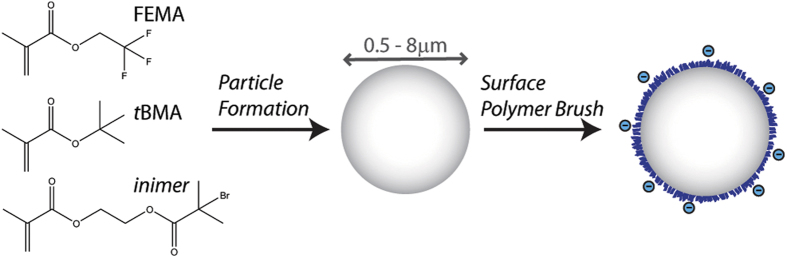
Schematic representation of the synthesis and surface modification of copolymer colloids by dispersion polymerization and ATRP.

**Figure 2:**
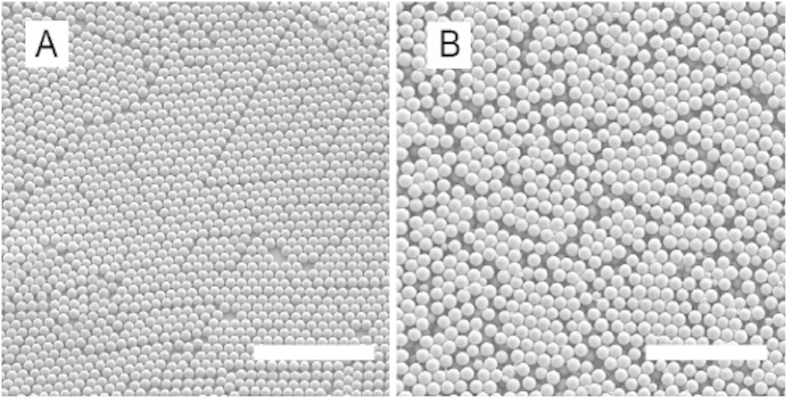
Scanning electron microscopy (SEM) image of particle with an (**A**) average diameter of 1.4 *μ*m (**B**) and an average diameter of 1.8 *μm*. Scale bar in both images is 20 *μm*.

**Figure 3:**
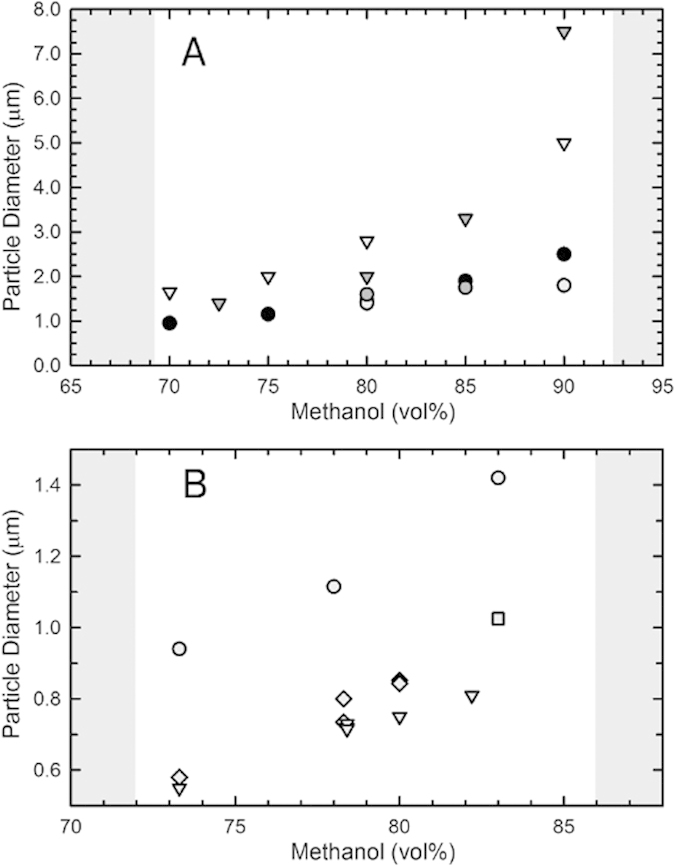
(**A**) PVP stabilized particle diameters with changes in co-solvent and inimer type, see Table in SI. 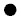 H_2_O, no inimer; 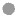 H_2_O, acrylate inimer; 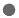 H_2_O, methacrylate inimer; 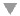 formamide, acrylate inimer; 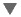 formamide, methacrylate inimer. (**B**) SPMA stabilized particle diameters with cosolvent, H_2_O, volume varied along with total monomer volume fraction, see Table in SI. 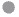 15vol% monomer; 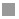 12.5vol% monomer; 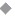 10vol% monomer; 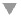 5vol% monomer. Reaction compositions within shaded regions yield only polymerized coagulum.

Here we develop a colloidal system which can be suspended in polar solvents, in which the refractive index and density can be controlled precisely, and whose interactions are tunable using controlled living radical polymerisation methods, illustrated in Fig. 1. Fluorinated methacrylate polymers, such as poly(trifluoroethyl methacrylate), have relatively low refractive indices, *n* ≤ 1.415, yet a very large density, *ρ* ∼ 1.538 g/ml. Conversely, aliphatic methacrylate polymers have densities that decreases with the length of the alkyl group: *ρ*∼ methyl methacrylate >ethyl methacrylate >t-butyl methacrylate, but exhibit relatively high refractive indices, *n*_*PMMA*_ ≈ 1.495. Combining these two types of monomers at different molar ratios yields a copolymer of which density and refractive index can be tuned; a similar strategy to tune the refractive index of the PMMA system was reported previously^29^. Here we choose a combination of trifluoroethyl methacrylate (TFEMA) and *tert*-butyl methacrylate (*t*BMA). Homopolymers of each exhibit the following properties: PTFEMA [*ρ* = 1.53 g/ml, *n* = 1.4185] and P*t*BMA [*ρ* = 1.022 g/ml, *n* = 1.4630]. The dispersion copolymerization of TFEMA and *t*BMA yields particles with a very low size polydispersity, typically CV ≤ 5% (Fig. 2, see SI). The particle size can be tuned precisely from *a* ∼ 0.55 *μ*m–8 *μ*m by changing the type and amount of cosolvent and by the monomer volume fraction charged during dispersion polymerization, see Fig. 3. At a ratio of 28:72 of TFEMA:*t*BMA, by volume, particles have a relatively low density, *ρ* = 1.16 g/ml, refractive index, *n* = 1.452, and high glass transition temperature, T_*g*_ ∼ 86 °C as measured by differential scanning calorimetry while in the suspending solution. This comonomer ratio is specifically chosen to refractive index and density match the colloids to a mixture of polar solvents, formamide and sulfolane.

**Figure 4:**
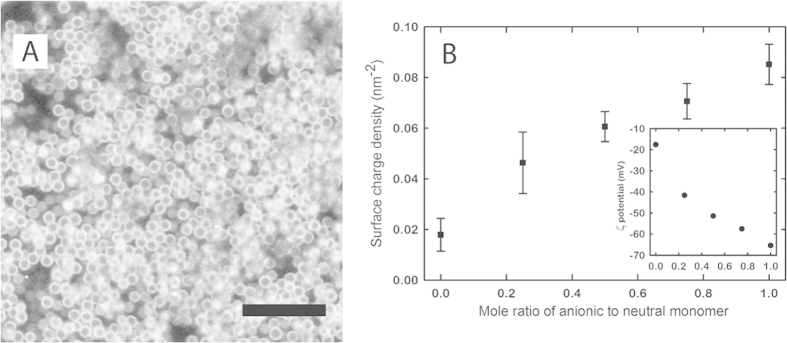
(**A**) 2D confocal microscopy image of non-fluorescently labeled particles with a fluorescent ‘halo’ indicative of a brush grown from the colloidal surface by ATRP. Scale bar is 10 *μm*. (**B**) Calculated surface charge density, using Eq. (1), after ATRP with different molar ratios of anionic to neutral monomer. Inset: *ζ* potential values measured in 10 mM TRIS, pH 7.5.

The phase behavior and dynamics of colloidal systems depends sensitively on the pair interactions between the particles. Tuning these interactions thus allows the creation of a wide variety of liquid and solid states. Typically, the interactions between particles results from moieties at the particle surface which originate from the synthesis, such as residual charges from initiators or stabilizers. Here we present a method which offers precise control of surface functionality in a separate post-synthesis modification step, using surface-initiated Atom Transfer Radical Polymerization^26^. During the dispersion polymerization of the particles, we covalently incorporate an ATRP initiator^30^. While the co-polymerisation of the initiator with the bulk of the particle polymer is random, the inimer itself remains soluble and thus is enriched at the particle surface where it remains accesible for the subsequent modification step. Including this functional monomer does not influence the particle size or polydispersity as shown in Fig. 3. To confirm that the ATRP initiator remains active after the dispersion polymerization, we grow a brush of a fluorescent monomer from the surface of the particles. While this brush thickness is well below the diffraction limit, a fluorescent halo is clearly distinguishable around each particle when imaged using confocal microscopy, indicating uniform surface modification despite the presence of a steric polymer layer, as shown in Fig. 4A.

Typically, the degree of polymerization of these brushes is determined by the molar ratio of monomer to surface initiator molecules, however, the precise number of ATRP initiators at the particle surface, and therefore the number of growing polymer chains, is unknown. Instead, we control the molecular weight of the growing chains by adding a sacrificial initiator to the bulk reaction at a concentration significantly higher than the maximum number of possible reactive groups present on the particle surface. Our approach has the additional benefit that polymerizations operated with a large excess of sacrificial initiator are less sensitive to inhibitors, impurities, and small procedure variations. We use this approach to prepare copolymer brushes of a neutral, dimethyl acrylamide, DMA, and anionic, sulfopropyl acrylamide, SPAm, at different molar ratios of the two monomers. In this way, the surface charge density, and thereby the colloidal interactions, can be carefully and reproducibility tuned. The surface charge density of these particles is calculated using measured *ζ*-potentials and using the following empirical relation^31^:


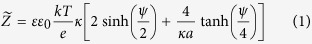


where 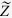 is the surface charge density, *ε* is the dielectric constant of the suspending fluid (∼82), *ε*_0_ is vacuum permittivity, *k* is the Boltzmann constant, T is the absolute temperature, *e* is the elementary charge, ***κ*** is the inverse Debye screening length, and 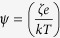. By adjusting the ratio of neutral to charged monomer, we control the final charge density on the particles, from neutral to highly charged, which is not possible with other colloidal systems while permitting refractive index and density matching (Fig. 4B).

Once the particles are formed, their surfaces modified, and a fluorescent label incorporated, these colloids may be used for confocal microscopy experiments only if the suspending fluid meets certain experimental criteria: non-volatile, high dielectric constant, and match both the refractive index and density of the colloids. Additionally, the fluid must not swell or soften the particle, which causes gradual degradation of the particles, changes in volume fraction and leaking of non-covalently bound fluorophore from the particle. All of these requirements are met by a choosing a specific comonomer ratio of the polymer which composes the particle, such that a 72:28 by volume mixture of formamide and sulfolane solution matches both the refractive index and density of the particles. The density difference between fluid and copolymer particle is minute, confirmed by the absence of visual signs of sedimentation or creaming even after centrifugation at 20,000 g for 48 hours. Moreover, this mixture does not plasticize the particles, as inferred by observing no fluorophore leaking into the suspending fluid, even after storage for over 2 years. Interestingly, this mixture of solvents has a dielectric constant of *ε* ∼ 82, nearly the same as that of water^32^.

**Figure 5:**
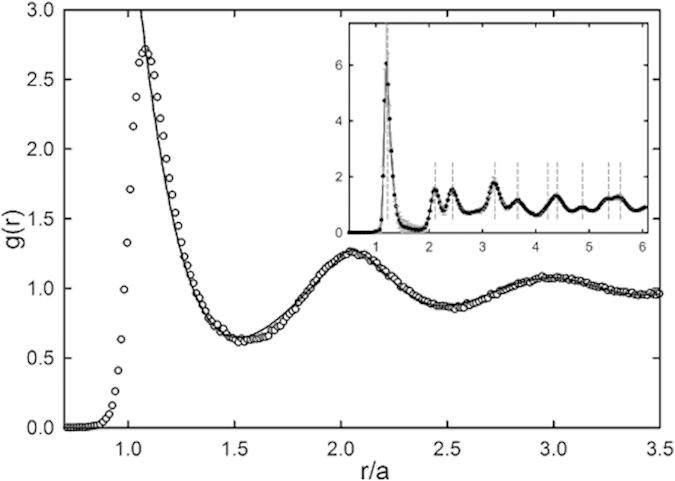
3D radial distribution function, g(r), normalized by particle radius, *a* from SEM. (o) nearly hard-sphere like interaction potential (*ϕ* ∼ 0.40, 50 mM NaCl); (solid line) hard-sphere behavior calculated using the Percus-Yevick approximation. Inset: (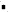) pair correlation function for the Wigner crystal (*ϕ* ∼ 0.40, ∼0 mM NaCl); dashed lines are hexagonal close packed positions. The nearest neighbor peak is shifted to *r*/*a* ∼ 1.24 indicative of a long ranged repulsive interaction potential.

This highly polar solvent mixture allows the dissolution of large concentrations of electrolyte to screen charge repulsion, rendering the interactions between the colloids hard-sphere like. We take a suspension of fluorescently-labeled colloids, modified with a copolymer brush of DMA and SPAm, at a volume fraction *ϕ* = 0.42 ± 2, and add 50 mM of sodium chloride to screen the surface charges. From confocal fluorescence microscopy images, we extract particle positions in three dimensions from which the radial distribution function, *g*(*r*), is computed. This pair correlation function in this fluid regime can be accurately described by the Perkus-Yevick closure approximation for pure hard spheres, see Fig. 5, confirming that interactions occur through volume exclusion alone. We note that, since particles are not swollen by either of the two solvents used here, volume fractions determined from particle counting in three-dimensional confocal microscopy and those determined through solvent evaporation with thermogravimetric analysis, or from sedimentation, are identical to within the noted experimental error^16^.

**Figure 6:**
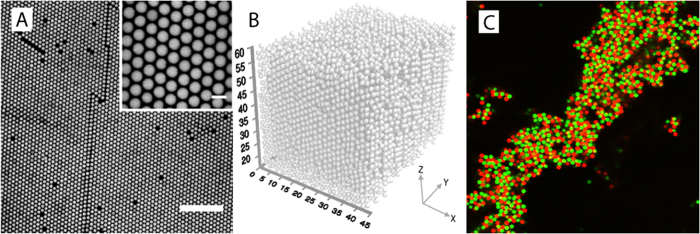
(**A**) 2D x–y confocal microscopy slices of a Wigner crystal; particles at *ϕ* ∼ 0.40 in deionized, refractive index and density matched solution. Scale bar is 20 *μm*. Inset: higher magnification, with a scale bar of 2 *μm*. (**B**) 3D particle reconstruction from particle locations; distances in microns, starting at 15 *μm* from the coverslip. (**C**) 2D confocal microscopy images of refractive index and density matched colloidal gels composed of 1.85 *μm* diameter particles with an anionic (green) and cationic (red) surface charge in ∼0 mM NaCl.

Interestingly, if we take the same colloidal particles at an identical volume fraction, and de-ionize the fluid mixture to remove any ionic species and impurities, the particles begin to interact through long-ranged electrostatic repulsions. The conductivity of formamide as received is 68 *μ*S/cm and that of sulfolane is 6.6 *μ*S/cm. After deionization with a mixed bed ion exchange resins, the conductivity decreases to 1.0 *μ*S/cm and 0.23 *μ*S/cm, respectively. While the screened sample at a volume fraction below the freezing limit for hard-spheres exhibited a fluid structure, the particles in this deionized refractive index- and density matching mixture crystallize due to the electrostatic repulsions, forming a so-called colloidal Wigner crystal (Fig. 6A,B), in which distinct and sharp peaks in the radial distribution function are observed (inset Fig. 5).

When the same particles, labeled with a yellow fluorescent dye, are mixed with particles from the same batch whose surface has instead been functionalized with a cationic polymer brush and contain a red fluorescent dye, charge attraction between the oppositely charged species gives rise to the formation of electrostatically-assembled gels (Fig. 6C). Such gels show distinctly different behavior from colloidal networks formed by aspecific aggregation in a single component dispersion^21^,^22^.

**Figure 7:**
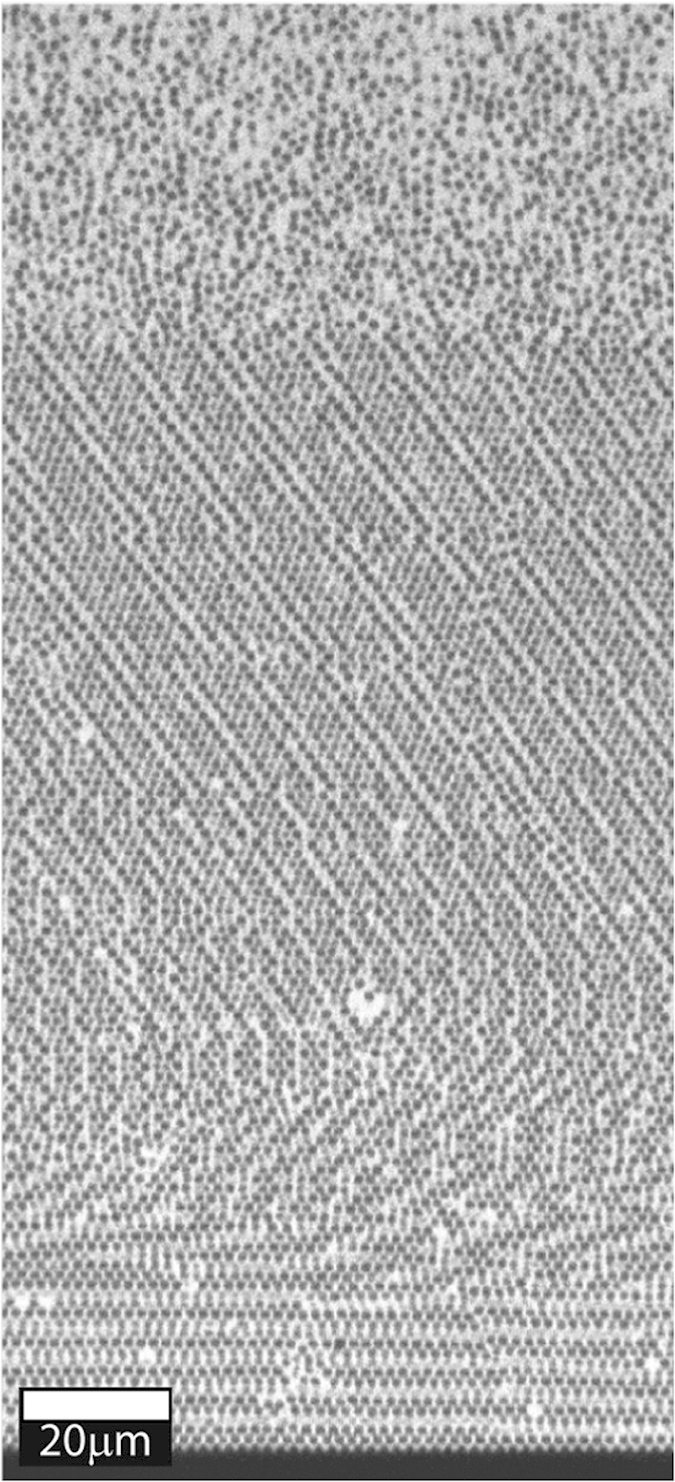
Deep XZ confocal slice of refractive index matched but density mismatched 1.65 *μm* diameter particles dispersed in formamide with 30 mM NaCl.

While this system of particles composed of 2 monomers, and a fluid mixture of only 2 components, allows almost perfect matching of the densities, certain experiments may require a controlled density mismatch, for example to templating of crystals on patterned substrates^11^,^12^,^13^, or to ascertain the equation of state.^33^ In the system we present here, minute changes to the copolymer ratio allows doing exactly this. These changes do not influence the particle size or polydispersity shown in Fig. 3. To showcase this we prepare particles which can be perfectly refractive index matched in pure formamide, but which exhibit a mild density mismatch of 0.077 g/cm^3^. A sample at *ϕ* ≈ 0.02 is equilibrated for 2 days, during which a crystalline sediment forms, a sharp and distinct crystal-fluid interface becomes apparent. The refractive index match allows us to image very deep into the sample, seen in the confocal microscopy image in Fig. 7 which shows a penetration depth of the excitation laser of well over 220 *μ*m into the sample, without significant optical aberrations even in the direction perpendicular to the confocal scanning plane.

Finally, we show that the same particles can be created in a core-shell variant, in which a fluorescent core is embedded in a non-fluorescent shell. Such core-shell architecture is particularly useful for studies of dense suspensions in which locating particle centroids with high accuracy can be difficult. Separating the fluorescent centers of the particles with a non-fluorescent shell of the same material is known to greatly enhance the resolution and accuracy of particle locating algorithms^27^.

**Figure 8:**
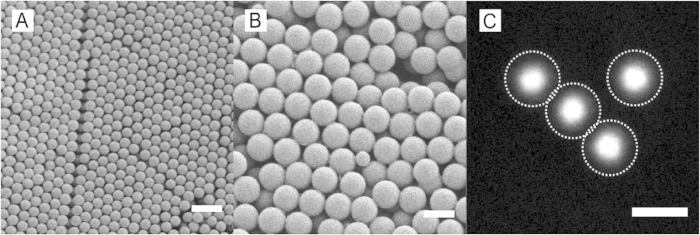
(**A**) SEM image of core particles, *a* ∼ 710 *nm* (**B**) SEM image of final particles, *a* ∼ 1.8 *μm* (**C**) 2D confocal microscopy image of the fluorescent core with shell outlines (dotted lines) determined by simultaneous bright-field microscopy, not shown. The single small particle seen in (**B**) is a core from (**A**) that was not encapsulated during shell polymerization. Scale bar in all images is 2 *μ*m.

We prepare core-shell particles by seeded-dispersion polymerization in the presence of cross-linked core particles in which a fluorescent dye is covalently attached to the polymer chains. These crosslinked cores are prepared using precipitation polymerization, which yields monodispersed particles with a clean surface free of dispersant or surfactant^34^ (Fig. 8A). These polymerizations are performed at low volume fractions of monomer; reactions in which the monomer concentration was increased to >5 vol%, and with crosslinker concentrations in excess of 2 vol%, produced an undesirably large number of dimers and trimers. Subsequently, a non-fluorescent shell is formed around the cross-linked core particles by a seeded dispersion polymerization. During polymerization, the cosolvent ratio determines the maximum particle size possible; secondary nucleation can be avoided by using low amounts of cosolvent. This results in high yields of monodisperse core-shell particles (Fig. 8B), whose surface can be functionalized using the same surface-initiated ATRP procedure as discussed above. Confocal imaging of these particles yields distinctly separated fluorescent centers, even when the particles are in direct contact (Fig. 8c).

## Conclusions

In this paper we present a complete method to produce monodisperse colloidal particles, whose refractive index and density can be tailored and whose interactions can be precisely tuned using surface-initiated growth of polymer brushes. The simultaneous matching of refractive index and density, in a non-volatile, non-hazardous and polar solvent, allows high resolution quantitative imaging of these systems with three-dimensional confocal microscopy. Moreover, by tuning the composition of the polymer brush at the surface, and controlling the ionic strength of the suspending medium, we tune the interactions from hard-sphere like, where volume exclusion alone governs their phase behavior, to long-ranged repulsive or attractive. The synthesis we describe uses ingredients which are readily available, and whose procedure is highly reproducible. This resolves many of the constraints imposed by commonly used experimental systems for exploring the physics and physical chemistry of colloidal dispersions. Moreover, due to the polarity of the suspending medium, our systems opens the way to the preparation of particles which exhibit specific interactions, for example using supramolecular motifs, that allow the study of directed colloidal self-assembly in three dimensions and in absence of gravitational stresses.

## Methods

All materials are purchased from Sigma-Aldrich and used as received, unless otherwise noted.

### Dispersion polymerization

Most particle polymerizations using poly-vinylpyrrolidone, PVP, as the steric stabilizer are conducted in a 200 ml round bottom flask tumbled in a heated glycerol bath by an overhead stirrer. Typical polymerization mixtures are given in the SI. A dispersion reaction proceeds as follows: methanol (90.0 ml), deionized water (10.0 ml), 2,2,2-trifluoroethyl methacrylate (2.8 ml, FEMA, SynQuest Laboratories), *t*-butyl methacrylate (7.2 ml, tBMA, TCI America) at a ratio of 28:72 by volume, polyvinylpyrrolidone (4.0 g, PVP K30), the *inimer* (2-(2-bromoisobutyryloxy) ethyl acrylate or 2-(2-bromoisobutyryloxy) ethyl methacrylate, 0.5 ml, see SI for synthesis protocol) and 0.1 g of 2,2′,-Azobis(2-methylpropionitrile) (AIBN) are added to the reaction flask. The flask is brought under vacuum and subsequently purged with nitrogen, which is repeated several times to obtain oxygen-free conditions. The reaction mixture is then slowly rotated at ≈75 rpm in a glycerol bath at 55 °C for 16 hours. The resulting particles are washed by repeated centrifugation and redispersion in a 1:1 water-methanol mixture. Polymerizations are invariant to total reaction volume; reaction volumes up to 1000 ml have been conducted with no changes to the final particle size and polydispersity.

### Core-shell particles

Cross-linked core particles are prepared using precipitation polymerization using a charged comonomer 3-sulfopropyl methacrylate (SPMA), conducted in a 200 ml round bottom flask equipped with a reflux condenser. Detailed description of the reaction mixtures are given in the SI. A typical polymerization reaction proceeds as follows: methanol (80.0 ml), deionized water (22.0 ml), 2,2,2-trifluoroethyl methacrylate (1.45 ml), *t*-butyl methacrylate (3.9 ml), SPMA potassium salt (0.055 g), acrylate inimer (0.100 ml, 2vol% to monomer), ethylene glycol dimethacrylate (0.107 g, 2vol% to monomer), a solution of fluorescent monomer, rhodamine B methacrylate or coumarin methacrylate (see SI for synthetic procedure, 1.0 ml, 2wt% in methanol) and AIBN (0.055 g) are added to the reaction flask. The reaction flask is then heated to reflux, without degassing, at ∼80 °C for 5 hours. After completion, the particles are washed by repeated centrifugation and redispersion (5X) in a 1:1 water-methanol mixture. Successful covalent attachment of fluorophores is verified by suspending crosslinked particles in tetrahydrofuran (THF) in which they maintain a spherical shape and strong fluorescence, with no fluorescence visible in the suspending solvent. A non-fluorescent shell is grown around the colloids using seeded dispersion polymerization, following the procedure described above. The thickness of the shell can be tuned by the ratio of fluorescent seed particles to total monomer.

### Surface functionalization

The *inimer* that is copolymerized during the dispersion polymerization allows for the use of ATRP to directly grow a polymer from the surface of the colloid^26^. To control the degree of polymerization of the growing polymer, a sacrificial ATRP initiator is added to the solution, assumed to be in large excess of the available surface *inimer* molecules^35^. This sacrificial initiator (PEGini) is synthesized identically to the acrylate inimer exchanging 2-hydroxyethyl acrylate for poly(ethylene glycol) methyl ether, *M*_*n*_ = 550. A typical surface-modification proceeds as follows: formamide (FM, 32 ml), water (26 ml), *PEGini* (1.25 ml, 2.26 mmoles), 2-acrylamido-2-methyl-1-propanesulfonic acid sodium salt (SPAm, 10.1 mmoles, 50wt% in H_2_O), dimethylacrylamide (DMA, 47.1 mmoles), 1,1,4,7,10,10-hexamethyltriethylenetetramine (HMTETA, 2.38 mmoles), Cu(II)Cl_2_ (1.13 mmoles) are added to a 200 ml flask containing the particle suspension (65 ml, 25% solids, in 2:1 H_2_O:FM). The molar ratio between monomer:*PEGini*:copper:ligand is [25:1:1:1.05]. The suspension is bubbled with nitrogen or argon for at least 20 minutes to remove dissolved oxygen, then 1.13 mmoles of Cu(I)Cl is added to the flask to initiate the polymerization and tumbled for several hours (≥ 6 hrs). A fluorescently-labelled polymer brush is prepared from fluorescein methacrylate 3:1 by mass of 2,2-bipyridyl:Cu(II)Br in 2:1 by volume FM:H_2_O. The reaction is initiated with 5 mg of ascorbic acid and allowed to proceed for 1 hour.

### Index and density matching

Particles are washed into deionized formamide (FM), with a conductivity of *σ* = 1.00 *μ*S. Deionized tetramethylsulfonone (SF, *σ* = 0.23 *μ*S) is added drop wise to a particle suspension at a volume fraction of *ϕ* ∼ 0.40 until the sample is transparent as judged visually. Density matching is verified by centrifuging the dispersion at 3000 *g* for 6 hours. The suspending fluid mixture is adjusted until no sedimentation or creaming is observed. By synthesizing the colloids from a specific volumetric ratio of monomers (FEMA:tBMA, 28:72), both refractive index and density are simultaneously matched with a mixture of FM and SF at 72:28 by volume. The final suspending fluid mixture has an estimated dielectric constant of *ε* ∼ 82^32^. A mixed bed ion exchange resin (Dowex Marathon MR-3) is added to further deionize the suspending fluid mixture to control charge interactions.

### Particle characterization

Particle sizes and polydispersities are measured from both brightfield microscopy and scanning electron microscopy (SEM, Zeiss Supra 55). We define the coefficient of variation, CV, as the standard deviation over the mean particle radius, 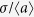. Refractive index and density matched samples are allowed to equilibrate for several hours in hermetically sealed sample chambers, after which 3D image stacks are recorded using a confocal fluorescence microscope (Leica SP5). Radial distribution functions are calculated using standard particle locating algorithms^36^. Zeta potentials are measured with a Malvern Zetasizer Nano ZS on dilute particle suspensions in 10 mM PIPES buffer at pH 7.0. The glass transition temperature of the copolymer particles in the index- and density-matching mixtures is measured with differential scanning calorimetry (TA Instruments, Q200).

## Additional Information

**How to cite this article**: Kodger, T. E. *et al.* Precise colloids with tunable interactions for confocal microscopy. *Sci. Rep.* **5**, 14635; doi: 10.1038/srep14635 (2015).

## References

[b1] Anderson V. J. & Lekkerkerker H. N. W. Insights into phase transition kinetics from colloid science. Nature 416, 811–815 (2002). 11976674 10.1038/416811a

[b2] Wang Z., Wang F., Peng Y., Zheng Z. & Han Y. Imaging the homogeneous nucleation during the melting of superheated colloidal crystals. Science 338, 87–90 (2012). 23042889 10.1126/science.1224763

[b3] Pertsinidis A. & Ling X. S. Diffusion of point defects in two-dimensional colloidal crystals. Nature 413, 147–150 (2001). 11557976 10.1038/35093077

[b4] Weeks E. R., Crocker J. C., Levitt A. C., Schofield A. & Weitz D. A. Three-dimensional direct imaging of structural relaxation near the colloidal glass transition. Science 287, 627–631 (2000). 10649991 10.1126/science.287.5453.627

[b5] Kegel W. K. & van Blaaderen A. Direct observation of dynamical heterogeneities in colloidal hard-sphere suspensions. Science 287, 290–293 (2000). 10634780 10.1126/science.287.5451.290

[b6] Aarts D. G. A. L. The interface in demixed colloidal polymer systems: wetting, waves and droplets. Soft Matter 3, 19–23 (2007). 10.1039/b608479f 32680188

[b7] Wang Y. *et al.* Colloids with valence and specific directional bonding. Nature 491, 51–55 (2012). 23128225 10.1038/nature11564

[b8] Zhu J. *et al.* Crystallization of hard-sphere colloids in microgravity. Nature 387, 883–885 (1997).

[b9] Royall C. P., Dzubiella J., Schmidt M. & van Blaaderen A. Nonequilibrium sedimentation of colloids on the particle scale. Physical Review Letters 98, 188304 (2007). 17501616 10.1103/PhysRevLett.98.188304

[b10] Schall P., Weitz D. A. & Spaepen F. Structural rearrangements that govern flow in colloidal glasses. Science 318, 1895–1899 (2007). 18096800 10.1126/science.1149308

[b11] Ramsteiner I. B., Jensen K. E., Weitz D. A. & Spaepen F. Experimental observations of the crystallization of hard-sphere colloidal particles by sedimentation onto flat and patterned surfaces. Physical Review E 79, 011403 (2009). 10.1103/PhysRevE.79.011403 19257031

[b12] Jensen K. E., Pennachio D., Recht D., Weitz D. A. & Spaepen F. Rapid growth of large, defect-free colloidal crystals. Soft Matter 9, 320–328 (2013).

[b13] van Blaaderen A., Ruel R. & Wiltzius P. Template-directed colloidal crystallization. Nature 385, 321–324 (1997).

[b14] Monch-Jorda A., Louis A. A. & Padding, J. T. Effects of interparticle attractions on colloidal sedimentation. Physical Review Letters 104, 068301 (2010). 20366858 10.1103/PhysRevLett.104.068301

[b15] Pusey P. N. & van Megen W. Phase behaviour of concentrated suspensions of nealy hard colloidal spheres. Nature 320, 340–342 (1986).

[b16] Royall C. P., Poon W. C. K. & Weeks E. R. In search of colloidal hard spheres. Soft Matter 9, 17 (2013).

[b17] Helden A. V. & Vrij A. Static light scattering of concentrated silica dispersions in apolar solvents. Journal of Colloid and Interface Science 320, 312–329 (1980).

[b18] Smith G. N. & Eastoe J. Controlling colloid charge in nonpolar liquids with surfactants. Physical Chemistry Chemical Physics 15, 424 (2013). 23187453 10.1039/c2cp42625k

[b19] Kanai T. *et al.* Crystallization and reentrant melting of charged colloids in nonpolar solvents. Phys. Rev. E 91, 030301 (2015). 10.1103/PhysRevE.91.030301 25871032

[b20] Yethiraj A. & van Blaaderen A. A colloidal model system with an interaction tunable from hard sphere to soft and dipolar. Nature 421, 513–517 (2003). 12556887 10.1038/nature01328

[b21] Russell E., Sprakel J., Kodger T. & Weitz D. Colloidal gelation of oppositely charged particles. Soft Matter 8, 8697 (2012).

[b22] Sprujit E. *et al.* Reversible assembly of oppositely charged hairy colloids in water. Soft Matter 7, 8281 (2011).

[b23] Go D., Kodger T. E., Sprakel J. & Kuehne A. J. C. Programmable co-assembly of oppositely charged microgels. Soft Matter 10, 8060–8065 (2014). 25169820 10.1039/c4sm01570c

[b24] Leunissen M. E. *et al.* Ionic colloidal crystals of oppositely charged particles. Nature 437, 235–240 (2005). 16148929 10.1038/nature03946

[b25] Bartlett P. & Campbell A. I. Three-dimensional binary superlattices of oppositely charged colloids. Physical Review Letters 95, 128302 (2005). 16197118 10.1103/PhysRevLett.95.128302

[b26] Perruchot C. *et al.* Synthesis of well-defined, polymer-grafted silica particles by aqueous atrp. Langmuir 17, 4479–4481 (2001).

[b27] Elsesser M. T., Hollingsworth A. D., Edmond K. V. & Pine D. J. Large core-shell poly(methyl methacrylate) colloidal clusters: Synthesis, characterization, and tracking. Langmuir 27, 917–927 (2011). 21190338 10.1021/la1034905

[b28] Dullens R. P. A., Claesson M., Derks D., van Blaaderen A. & Kegel W. K. Monodisperse core-shell poly(methyl methacrylate) latex colloids. Langmuir 19, 5964–5966 (2003).

[b29] Underwood S. M. & van Megen W. Refractive index variation in nonaqueous sterically stabilized copolymer particles. Colloid Polym Sci 274, 1072–1080 (1996).

[b30] Matyjaszewski K., Gaynor S. G., Kulfan A. & Podwika M. Preparation of hyperbranched polyacrylates by atom transfer radical polymerization. 1.acrylic ab* monomers in atom transfer radical polymerizations. Macromolecules 30, 5192–5194 (1997).

[b31] Loeb A., Overbeek J. T. G. & Wiersema P. The electrical double layer around a spherical colloidal particles. MIT Press (1961).

[b32] Jouyban A., Soltanpourb S. & Chanc H.-K. A simple relationship between dielectric constant of mixed solvents with solvent composition and temperature. International Journal of Pharmaceutics 269, 353–360 (2004). 14706247 10.1016/j.ijpharm.2003.09.010

[b33] Piazza R., Bellini T. & Degiorgio V. Equilibrium sedimentation profiles of screened charged colloids: A test of the hard-sphere equation of state. Physical Review Letters 71, 4267–4270 (1993). 10055199 10.1103/PhysRevLett.71.4267

[b34] Zhang F., Cao L. & Yang W. Preparation of monodisperse and anion-charged polystyrene microspheres stabilized with polymerizable sodium styrene sulfonate by dispersion polymerization. Macromolecular Chemistry and Physics 211, 744–751 (2010).

[b35] von Werna T. W. *et al.* A versatile method for tubing the chemistry and size of nanoscopic features by living free radical polymerization. Journal of the American Chemical Society 125, 3831 (2003). 12656616 10.1021/ja028866n

[b36] Gao Y. & Kilfoil M. Accurate detection and complete tracking of large populations of features in three dimensions. Optics Express 17, 4685 (2009). 19293898 10.1364/oe.17.004685

